# ShK‐modified UCMSCs Inhibit M1‐Like Macrophage Polarization and Alleviate Osteoarthritis Progression via PI3K/Akt Axis

**DOI:** 10.1002/advs.202406822

**Published:** 2024-12-25

**Authors:** Wenshu Wu, Xueying An, Wang Gong, Lin Yang, Na Liu, Bin Liu, Baosheng Guo, Qing Jiang, Lan Li

**Affiliations:** ^1^ Division of Sports Medicine and Adult Reconstructive Surgery Department of Orthopedic Surgery Nanjing Drum Tower Hospital, Affiliated Hospital of Medical School, Nanjing University 321 Zhongshan Road Nanjing Jiangsu 210008 P. R. China; ^2^ State Key Laboratory of Pharmaceutical Biotechnology Nanjing University 22 Hankou Road Nanjing Jiangsu 210093 P. R. China; ^3^ Branch of National Clinical Research Center for Orthopedics Sports Medicine and Rehabilitation 321 Zhongshan Road Nanjing Jiangsu 210008 P. R. China; ^4^ Institute of Medical 3D Printing Nanjing University Nanjing 211166 P. R. China; ^5^ Jiangsu Engineering Research Center for 3D Bioprinting 321 Zhongshan Road Nanjing 210000 P. R. China; ^6^ Department of Sports Medicine and Adult Reconstructive Surgery Nanjing Drum Tower Hospital Clinical College of Nanjing Medical University Nanjing 210008 P.R. China

**Keywords:** Kv1.3, Macrophage, Osteoarthritis, UCMSC

## Abstract

The potassium channel Kv1.3 plays an important role in regulating immune cell functions in many inflammatory diseases whereas rarely in osteoarthritis (OA). Here, it is demonstrated that the Kv1.3 of macrophages is upregulated in response to LPS stimulation, as well as in human OA synovium samples than non‐OA. Administration of Stichodactyla toxin (ShK), a Kv1.3 blocker, significantly inhibited cartilage degeneration and synovial inflammation in animal models of OA in vivo by inhibiting M1 macrophage polarization and reducing the production of inflammatory factors. In this study, a transgenically engineered human umbilical cord mesenchymal stem cell (UCMSC) delivery system is developed that secreted a peptide ShK, a Kv1.3 potassium blocker, into the knee articular cavity. Collectively, the results identified Kv1.3 as a potential therapeutic target for OA and demonstrated the efficacy of using ShK transgenic engineered UCMSCs as a delivery for the peptide in OA treatment.

## Graphical abstract

Bioengineered UCMSCs are developed that enabled the production of a peptide secretion, ShK. The secreted peptide is functional in blocking Kv1.3 channels and suppressing the activation of macrophages in vitro and in vivo, which can provide potential treatments to halt osteoarthritis progression. The strategy provided a new idea for engineered stem cell therapy for osteoarthritis.

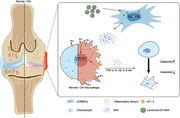

## 1 Introduction

Knee osteoarthritis (OA) is the most common degenerative joint disorder affecting an estimated 654 million people,^[^ ^1^ ^]^ which has significantly reduced the human quality of life and exacerbated the socioeconomic burden.^[^ ^2^ ^]^ Typical symptoms of OA are mainly pain, joint stiffness, and dysfunction. The present therapeutic strategy focuses on relieving pain symptoms and improving inflammation, such as palliative OA drugs diacerein, glucosamine, nonsteroidal anti‐inflammatory drugs (NSAIDs), and analgesics. However, due to the heterogeneity of the pathologic mechanisms of OA, current pharmacologic treatments that focus on symptomatic relief are not satisfactory. The pathogenesis of OA is still lacking and new therapeutic strategies need to be developed.^[^ ^3^ ^]^ Accumulating evidence indicates that synovial inflammation is a major contributor to OA,^[^ ^4^ ^]^ and M1‐like synovial macrophages, which an activated macrophages, are dramatically associated with joint inflammation and play critical roles in OA progression.^[^ ^5^ ^]^ In the progression of OA, abundant cells infiltrate the proliferative synovial tissue, macrophages accumulate in the synovial intimal lining pathologically, which is the major type of immune cells in OA different from rheumatoid arthritis major in T cells.^[^ ^6^ ^]^ Therefore, targeting the pathogenic mechanisms of M1‐like synovial macrophages and developing effective therapeutic agents for alleviating OA progression still need to be further explored.

Kv1.3 is highly expressed in immune cells and plays a key role in immune responses and inflammation, particularly in autoimmune diseases and chronic inflammatory conditions.^[^ ^7^ ^]^ Overexpression of Kv1.3 may lead to its overactivation, inducing macrophage M1 polarization, enhanced expression of IL‐6 and TNF‐α, and increased phosphorylation levels of ERK1/2 and NF‐κB in macrophages.^[^ ^8^ ^]^ Several studies have indicated that blocking Kv1.3 channels inhibits macrophage activation and attenuates inflammation levels.^[^ ^8^, ^9^ ^]^ While other inhibitors are limited by their own characteristics like nonbiological foreign chemicals, too large molecular weight to modify biologically, and so on. Stichodactyla toxin (ShK) is a 35‐residue basic peptide that was first found in a sea anemone *Stichodactyla helianthus*. It blocks Kv1.3 highly selectively and its analogs have been used as a therapeutic for autoimmune diseases.^[^ ^10^ ^]^ ShK is highly homologous to a domain of MMP23, and is likely recognized by the immune system as a self‐peptide, which has been successfully used in genetically engineered probiotic delivery systems.^[^ ^11^ ^]^ However, there is no evidence to indicate whether aberrantly Kv1.3 expression in activated M1‐like macrophages and OA progression. On the other hand, the current Kv1.3 inhibitors are peptides that have challenges such as high extraction costs, low uptake efficiency in vivo, easy degradation, etc.^[^ ^12^ ^]^ It is necessary to adopt in situ controlled drug release strategies to overcome the above problems.

Natural cells have been developed as carriers for therapeutic drugs due to their inherent ability to respond to the dynamic environment. In contrast, free proteins and therapeutic drugs typically provoke an immune system response, leading to their rapid removal from the bloodstream.^[^ ^13^ ^]^ Compared with synthetic materials, the living cells have greater biodegradability and biocompatibility.^[^ ^14^ ^]^ Human umbilical cord mesenchymal stem cells (UCMSCs) have gained increasing popularity in MSC‐based therapies due to their easy accessibility, high expansion capacity, low immunogenicity, and their regenerative, immunomodulatory, and anti‐inflammatory properties.^[^ ^15^ ^]^ Additionally, clinical trials based on MSCs have been conducted and have revealed promising therapeutic effects in different diseases including type 2 diabetes mellitus, refractory immune thrombocytopenia, psoriasis, OA, and so on.^[^ ^15^, ^16^ ^]^ Also, due to the high plasticity of stem cells, they can act as “Active Drug Factories” to consistently produce target peptides through transgenic technology.

In this study, we found that abnormally elevated expression of Kv1.3 in M1‐like synovial macrophages in OA contributes to the OA progression. Mechanistically, blocking Kv1.3 effectively inhibited macrophage M1 polarization and inflammatory release via the PI3K/Akt axis. Then we constructed the bioengineered UCMSCs to secrete ShK by transfecting UCMSCs with a plasmid overexpressing ShK, and the secreted peptide blocked Kv1.3 channels and suppressed the activation of macrophages in vitro and in vivo, thus inhibiting OA progression. To summarize, in this study developed a natural living functionalized carrier to manipulate macrophage polarization and remodel the disordered joint environment in OA. Our results provided new insights into the engineered stem cell therapy for OA.

## 2 Results

### 2.1 Kv1.3 is Upregulated in M1 Macrophages from the Synovium of OA than non‐OA

**Figure 1:**
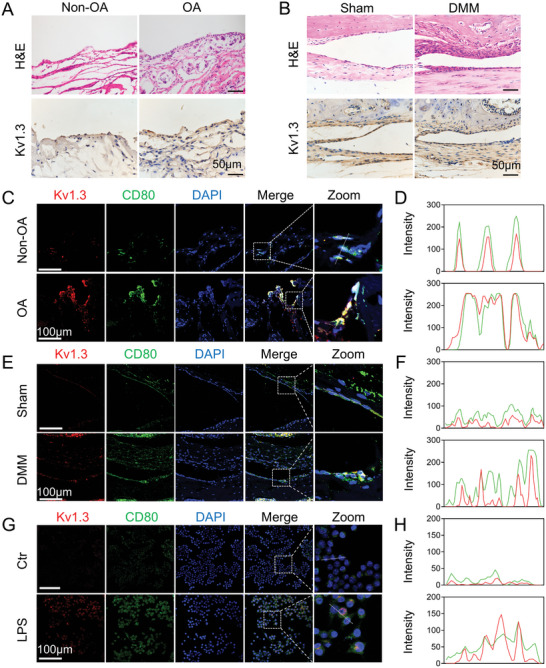
Kv1.3 is highly expressed in M1 macrophage in the synovium of OA patients and mice experimental OA models. A,B) Representative images of H&E staining and immunohistochemistry staining of Kv1.3 from patients’ synovium with or without OA, and mice with or without DMM operation after 8 weeks. Scale bar, 50 µm. C,D) Immunofluorescence staining of Kv1.3 (red) and CD80 (green) for synovium from OA or non‐OA samples. Scale bar, 100 µm. E,F) Immunofluorescence staining of Kv1.3 (pink) and CD80 (green) for synovium 8 weeks after sham or DMM operation. Scale bar, 100 µm. G,H) Representative images of immunofluorescence co‐staining of M1 marker CD80 (green) and Kv1.3 (red) in RAW264.7 macrophages. Scale bar, 100 µm. Data are means ± SD. Student's two‐tailed *t* test, ns, not significant *p* ≥ 0.05; ^*^ *p* < 0.05; ^**^ *p* < 0.01; ^***^ *p* < 0.001.

To investigate whether Kv1.3 channels get involved in OA progression, we first examined the expression of Kv1.3 in the synovium of OA patients and mouse OA models. The H&E staining obviously showed that the OA synovium was marked in synovial hyperplasia characterized by abundant cell infiltration whereas the non‐OA was not (**Figure** **1**A). Similar differences could be observed between the sham and DMM models (Figure 1B). We further also found that the proportion of Kv1.3 positive cells was greater in the articular synovium and significantly higher in the OA synovium than the non‐OA synovium from both human and mouse specimens (Figure 1A,B).

Next, to determine whether Kv1.3 expresses in the M1 macrophage, we examined the expression of Kv1.3 and M1 macrophage markers in the synovium of clinical samples, confirmed by IF co‐staining for Kv1.3 and CD80, a marker of M1 polarization macrophages. The colocalization of CD80 and Kv1.3 in the synovium of human samples was observed, suggesting that Kv1.3 was present in the M1 macrophages (Figure 1C,D). The double‐positive immunofluorescence (IF) staining of mouse synovium also revealed the expression of Kv1.3 in M1 macrophages in vivo. In vitro, the upregulated expression of Kv1.3 after LPS stimulation was also observed from the IF and WB results (Figure 1G,H and Figure S1A, Supporting Information) in RAW264.7 cells, a mouse macrophage line. These findings indicate an accumulation of M1 macrophages in the OA synovium, along with elevated levels of Kv1.3 expression during OA progression.

### 2.2 Kv1.3 Suppressor ShK Inhibits ROS and M1 Polarization in RAW264.7 Macrophages via the PI3K/Akt Axis

**Figure 2:**
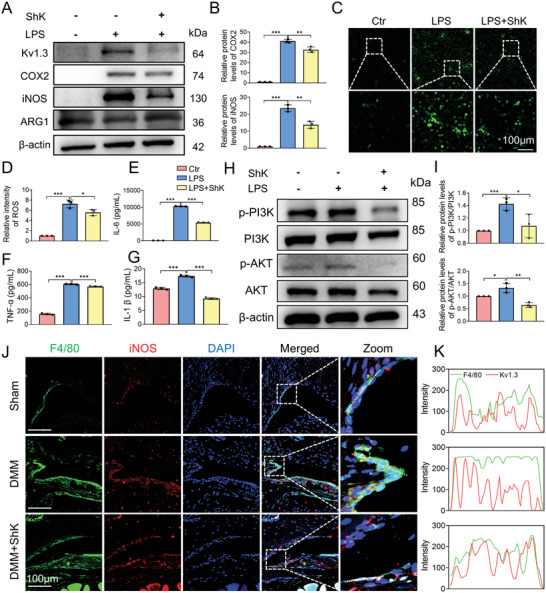
Kv1.3 suppressor ShK effectively inhibits ROS and M1 polarization via the PI3K/Akt axis. A,B) Western blot and quantitative analysis of Kv 1.3, iNOS, COX2 and ARG1 proteins in RAW264.7 macrophages treated with LPS with or without ShK for 24 h. C,D) Representative images of ROS in RAW264.7 macrophages pretreated with ShK (100 nm) with or without LPS (50 ng mL^−1^) in the 24 h. Scale bar, 100 µm. (E–G) ELISA analysis of IL‐1𝛽, IL‐6 and TNF‐𝛼 in supernatant of LPS treated RAW264.7 cells with or without ShK. (H‐I) Western blot analysis and quantification of p‐PI3K, PI3K, p‐AKT and AKT RAW264.7 in LPS treated macrophages with or without ShK. J,K) Representative immunofluorescence staining of macrophage marker F4/80 (green) and M1 polarization marker iNOS (red) in synovium 8 weeks after sham or DMM operation. Scale bar, 100 µm. Data are means ± SD. Student's two‐tailed *t* test, ns, not significant *p* ≥ 0.05; ^*^ *p* < 0.05; ^**^ *p* < 0.01; ^***^ *p* < 0.001.

To elucidate the mechanism of Kv1.3 in the M1 macrophage polarization, we used LPS‐treated RAW264.7 cells to induce M1 macrophage polarization. The protein levels of classical inflammatory factors, such as iNOS and COX2 increased after LPS treated compared to the control. ShK treatment could reverse these whereas the ARG1 (M2 marker) decreased and reversed after ShK treatment (**Figure** **2**A,B). Consistent with the IF result in Figure 1G, the protein expression of Kv1.3 was upregulated in LPS‐induced RAW264.7 cells and reversed by ShK treatment (Figure 2A). The proportion of cells positive for CD80 (M1 macrophage markers) was increased while reversed by inhibition of Kv1.3 by ShK and the proportion of CD163 positive cells (M2 macrophage markers) showed the contrary tendency (Figure S2A, Supporting Information). The ratio of iNOS/ARG1 protein expression in RAW264.7 cells was consistent with the IF result (Figure S2A,B, Supporting Information). LPS‐induced accumulation of ROS in RAW264.7 macrophages was significantly inhibited by ShK treatment (Figure 2C,D). The ELISA results revealed that increased secretory IL‐1𝛽, IL‐6 and TNF‐𝛼 in supernatant of LPS treated RAW264.7 cells was reversed after ShK treated (Figure 2E–G). Since inhibiting the Kv1.3 channel can prevent the activation of the PI3K/Akt pathway,^[^ ^17^ ^]^ which modulates the transcription of IL‐1β, IL‐6, TNF‐α and so on, we examined whether ShK mediated regulation of PI3K/Akt pathway. Western blot analysis revealed that ShK significantly reduced the phosphorylation levels of PI3K and Akt (Figures 2H,I). At the mRNA level, LPS stimulation notably increased the expression of *Il1b, Il6, Tnf, Nlrp3*, and *Nos2*. However, inhibiting Kv1.3 by ShK could reverse the increase of inflammatory factors at mRNA and protein levels in LPS‐treated RAW264.7 macrophages. Furthermore, intra‐articular administration of ShK could attenuate synovial hyperplasia and macrophage infiltration evidenced by immunofluorescence staining (Figure 2J,K). The proportion of CD80 positives cells was also significantly increased in the synovium of OA models and reversed by ShK treatment while the proportion of CD163 positive cells showed no significant difference among each group (Figure S2C,D, Supporting Information). These results suggest that the Kv1.3 suppressor ShK alleviates DMM‐induced cartilage erosion by inhibiting M1 polarization macrophage‐mediated activation in vitro.

### 2.3 Inhibition of Kv1.3 Effectively Inhibits OA Progression In Vitro and In Vivo

**Figure 3:**
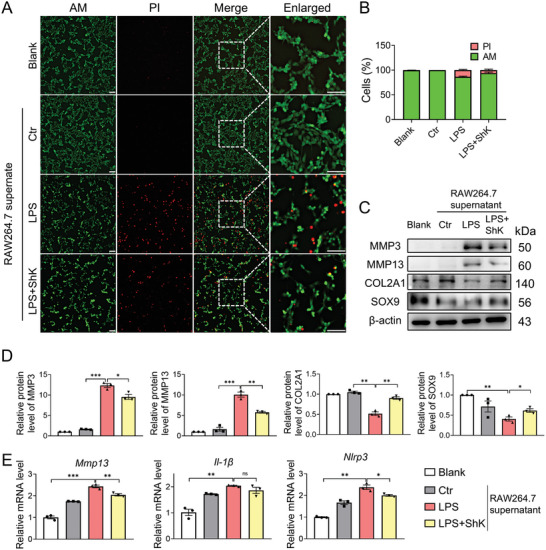
Inhibition of Kv1.3 inhibits OA progression in vitro. A,B) Representative images of live‐dead staining for ATDC5 cells after coculture with RAW264.7 cells with or without ShK treated after LPS stimulation. Scale bar, 100 µm. C,D) Western blot analysis and quantification of MMP3, MMP13, COL2A1 and SOX9 in ADTC chondrocytes after added supernate from LPS‐treated RAW264.7 macrophages with or without ShK. E) RT‐qPCR for messenger RNA of *Mmp13, Il1b, and Nlrp3* in chondrocytes in ADTC chondrocytes after added supernatant from LPS‐treated RAW264.7 macrophages with or without ShK. Data are means ± SD. Student's two‐tailed *t* test, ns, not significant *p* ≥ 0.05; ^*^ *p* < 0.05; ^**^ *p* < 0.01; ^***^ *p* < 0.001.

The main pathological feature of OA is cartilage degeneration characterized by the imbalance between excessive catabolism and insufficient or decreased anabolism.^[^ ^18^ ^]^ Subsequently, the medium from RAW264.7 cells was added to the ADTC5 chondrocytes. The supernatant medium from RAW264.7 cells treated with LPS induced an inflammatory phenotype in ADTC5 chondrocytes. The Calcein AM and propidium iodide (PI) staining (live/dead staining) revealed a decrease in the ratio of live to dead cells, demonstrating that ShK pretreatment of macrophages significantly alleviates the detrimental effects of LPS on cocultured chondrocytes (**Figure** **3**A,B). We also found that the medium from ShK‐treated RAW264.7 macrophages showed chondroprotective effects evidenced by western blot as decreased catabolism markers such as MMP3, MMP13 and reversed anabolism proteins like COL2A1 and SOX9 (Figure 3C–E). Taken together, these findings indicate that ShK can improve the OA‐like phenotype in chondrocytes cocultured with LPS‐pretreated RAW264.7 macrophages.

**Figure 4:**
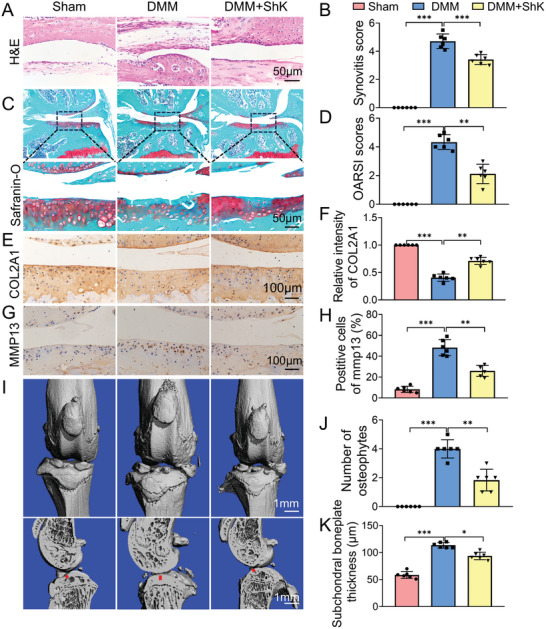
Kv1.3 suppressor ShK inhibits OA progression in vivo. A,B) Representative images of H&E staining of knee joint synovium and quantitative analysis of synovitis score. (n = 6). Scale bar, 50 µm. C,D) Representative images of safranin‐O/fast green staining of joint cartilage areas from sham, DMM and ShK‐treated DMM mice and OARSI scores were on the right side. (n = 6). Scale bar, 50 µm. E–H) Representative images of immunohistochemistry staining of COL2A1 and MMP13 for joint chondrocytes. (n = 6). Scale bar, 100 µm. I–K) 3D reconstructed images of knee joints and sagittal view of the medial joint compartment visualizing subchondral bone changes; a red line marks subchondral plate thickness. (n = 6) Scale bar, 1mm. Data presented as mean ± SD. ^**^ *p* < 0.01, ^*^ *p* < 0.05, ns, not significant, versus the indicated groups, Student's *t*‐test.

To determine whether Kv1.3 joins to regulate the macrophage polarization in OA, we took an intra‐articular injection of a Kv1.3 antagonist, ShK, in the mouse OA model. The H&E stained histological sections of the DMM group presented synovial hyperplasia characterized by the infiltration of abundant macrophages into the synovium, which was absent in the sections of the sham group (**Figure** **4**A,B). 8 weeks after DMM operation, the articular cartilage in the DMM group was notably thinner compared to the sham group, with larger surface fibrillation, increased loss of cartilage extracellular matrix, and more hypertrophic chondrocytes observed. However, following intra‐articular injection of ShK, we recorded improved Osteoarthritis Research Society International (OARSI) scores, increased extracellular matrix, fewer apoptotic chondrocytes and a smoother cartilage surface in the ShK treated group compared to the OA group (Figure 4C,D). IHC staining of the articular sections revealed a decrease in COL2A1 levels and an increase in the levels of matrix‐degrading enzyme MMP13 in the DMM group (Figure 4E–H). Furthermore, we assessed osteophyte formation using micro‐computed tomography (micro‐CT). 3D reconstructions of the articular samples from mice revealed increased joint mineralization, larger periarticular osteophytes, rougher tibial and femoral surfaces, and narrowed articular spaces, compared to the sham group. However, these pathological changes were ameliorated in ShK‐treated mice (Figure 4I–K). Taken together, these data showed that blocking the Kv1.3 channel could inhibit macrophage‐releasing inflammatory factors to alleviate DMM‐induced cartilage erosion and synovitis in vivo with negligible cytotoxic effects in major organs (Figure S4, Supporting Information).

### 2.4 Preparation and Characteristics of Engineered UCMSCs

**Figure 5:**
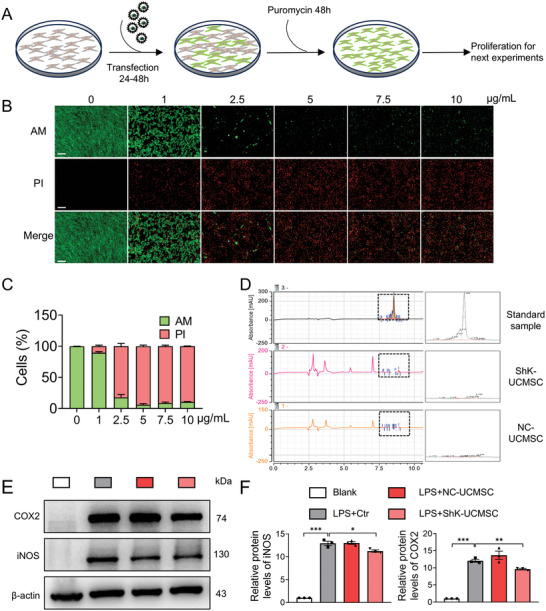
Preparation and characteristics of engineered UCMSCs. A) Experiment design diagram of transfection and screening of engineered UCMSCs. B) Representative images of Live‐dead staining staining of UCMSCs after screening by different concentrations of puromycin Scale bar, 100 µm. D) HPLC of supernatant from control or ShK‐UCMSCs after freeze‐drying and redissolved by the mobile phase. Detection is at 220 nm. E,F) Western blot analysis and quantification of COX2 and iNOS in LPS treated RAW264.7 macrophages added with supernate from with NC‐UCMSCs or ShK‐UCMSCs.Data are means ± SD. Student's two‐tailed *t* test, ns, not significant *p* ≥ 0.05; ^*^ *p* < 0.05; ^**^ *p* < 0.01; ^***^ *p* < 0.001.

To generate UCMSCs capable of secreting ShK, we transfected UCMSCs with lentivirus to overexpress ShK, and a schematic diagram of the experimental design for transfection is shown in **Figure** **5**A. The UCMSCs transfected with vehicle control lentivirus expressed green fluorescent protein (GFP), whereas the UCMSCs transfected with ShK overexpressing lentivirus did not express GFP (Figure S5, Supporting Information). Next, to obtain a high proportion of stable metastatic cells, we added puromycin (1µg mL^−1^) to screen for 48 h after transfection 48 h and exchanged normal culture medium to expand UCMSCs (ShK‐UCMSCs) for subsequent experiments (Figure 5B,C). Then, to evaluate the presence of active peptide ShK, we froze the supernatants by vacuum freeze–drying technology and detected the characteristic absorption peak by high‐performance liquid chromatography (HPLC) (Figure 5D). To further investigate whether the secreted ShK from ShK‐UCMSCs can inhibit Kv1.3 channels on RAW264.7cells, we cocultured LPS pretreated RAW264.7 macrophages with vehicle control vel‐UCMSCs or ShK‐UCMSCs. The western blot results indicated that compared to the vehicle control, ShK‐UCMSCs could reduce the production of inflammatory factors like COX2 and iNOS in RAW264.7 macrophages response to LPS stimulation (Figure 5E,F). Together, these results show that engineered UCMSCs can successfully secret functional ShK, which plays a similar role in inhibiting the production of inflammatory factors from M1 macrophages.

### 2.5 Engineered UCMSCs Attenuate OA Phenotype In Vivo

**Figure 6:**
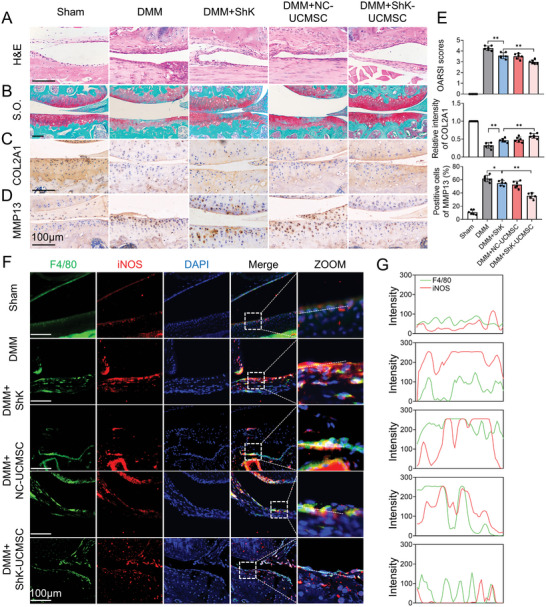
Engineered UCMSCs attenuate OA phenotype by inhibiting M1 macrophage polarization. A) Representative images of H&E staining of knee joint synovium. Scale bar,100 µm. B) Representative images of safranin‐O/fast green staining of joint cartilage from sham, DMM, DMM+ShK, DMM+NC‐UCMSC and DMM+ShK‐UCMSC mice. Scale bar,50 µm. C,D) Representative images of immunohistochemistry staining of COL2A1 and MMP13 for joint chondrocytes. (n = 6). Scale bar, 100 µm. E) OARSI scores and quantification of intensity of IHC in (C) and (D) using ImageJ. (n = 6). F) Immunofluorescence staining of iNOS (red) and F4/80 (green) for synovium from sham or DMM group. (n = 6). Scale bar, 100 µm. G) Quantification of intensity of IF staining in (F) using ImageJ. Data presented as mean ± SD. ^**^ *P* < 0.01, ^*^ *P* < 0.05, ns, not significant, versus the indicated groups, Student's *t*‐test.

**Figure 7:**
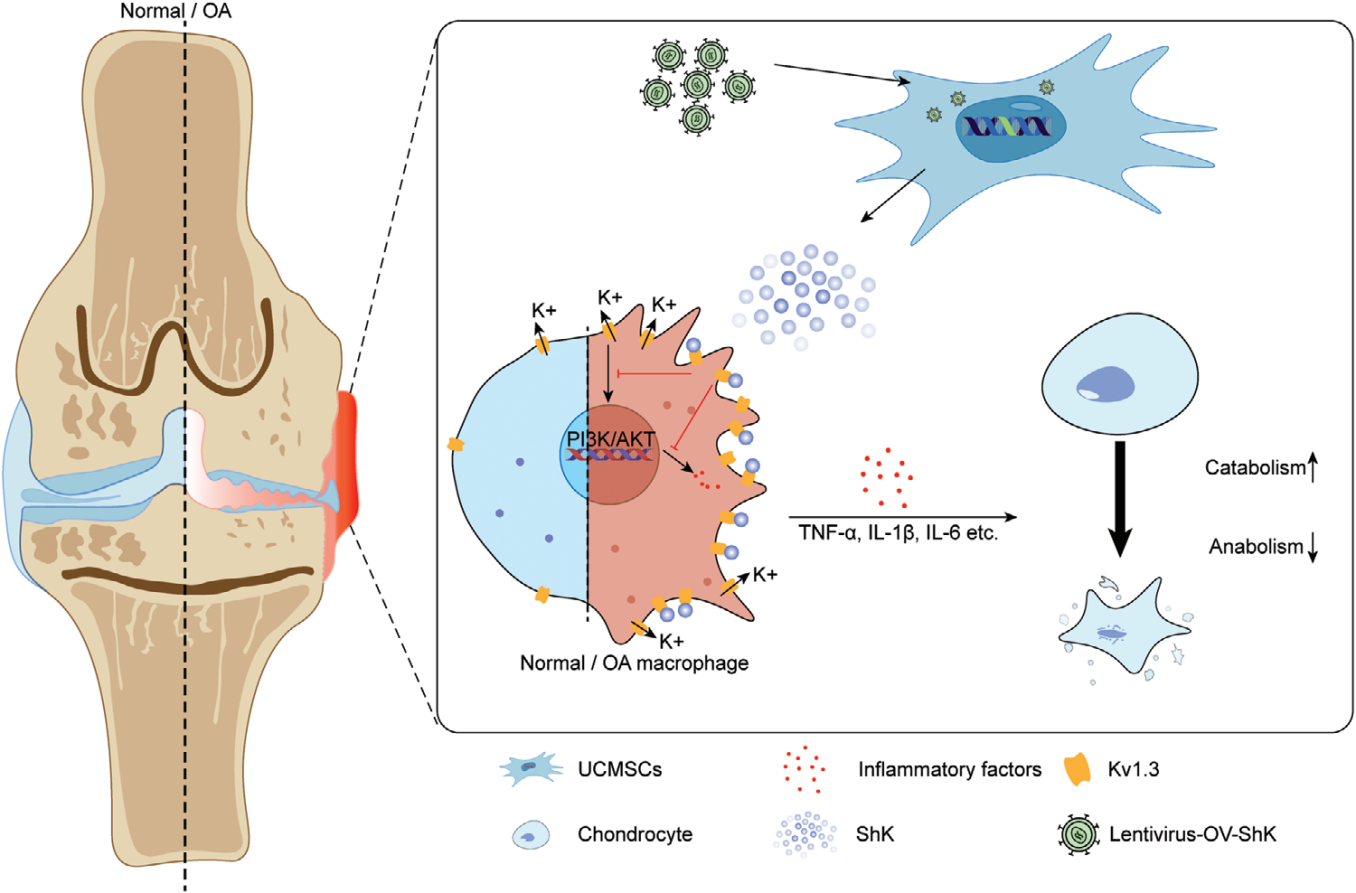
The scheme for ShK‐modified UCMSCs inhibit M1‐like macrophage polarization and alleviate osteoarthritis progression via PI3K/Akt axis.

To investigate the effects of the engineered ShK‐UCMSCs on OA progression in vivo, we performed intra‐articular injection of 10 µL volume PBS containing the vehicle control NC‐UCMSCs or ShK‐UCMSCs 10000 cells per mL, into knee joint cavities once every week for 8 weeks. We divided the experimental mice into the sham, PBS+DMM (DMM), ShK+DMM (ShK), vehicle control‐UCMSC+ DMM (NC‐UCMSC) and ShK‐UCMSC+DMM (ShK‐UCMSC) groups. The fluorescence signal from UCMSCs pretreated with fluorochrome DiR could last for ≈6–7 days detected by a small animal imaging system (Figure S6, Supporting Information). The H&E histological staining showed that the synovitis and macrophage infiltration were less common in the ShK‐UCMSC group than the DMM and Vel‐UCMSC groups (**Figure** **6**A). After 8‐week treatment, the cartilage erosion in the ShK‐UCMSC group presented a more regular surface and thicker articular cartilage while those in the PBS and Vel‐UCMSC groups showed poor surface regularity, thinner cartilage and abnormal distribution of chondrocytes (Figure 6B). Consistent with previous results, IHC staining of the articular sections was observed to reduce COL2A1 and increase matrix‐degrading enzyme MMP13 in the DMM group (Figure 6C–E). In addition, we found that iNOS was considerably low in F4/80 positive cells in DMM‐induced OA models (Figure 6F,G). Overall, ShK‐UCMSC group exhibited a better therapeutic effect than the ShK group characterized by less cartilage erosion, lower levels of catabolism marker MMP13, higher levels of anabolism marker COL2A1, and lower infiltration of M1 macrophage. Taken together, these results show that the application of ShK‐UCMSCs is more effective than the administration ShK alone in treating OA (**Figure** **7**).

## 3 Discussion

In this study, we showed that M1 macrophages accumulated in the OA synovium, along with elevated levels of Kv1.3 expression during OA progression. Inhibition of Kv1.3 through ShK effectively inhibits ROS production and M1 polarization by via PI3/Akt pathway in RAW264.7 macrophages. Considering that most kinds of peptide drugs require parenteral administration due to low oral bioavailability and the long‐term and repetitive injections in some chronic diseases could reduce patient compliance and lead to worse outcomes.^[^ ^19^ ^]^ We developed bioengineered UCMSCs that enabled the secretion of a peptide, ShK. The secreted peptide was effective in blocking Kv1.3 channels and inhibiting macrophage activation both in vitro and in vivo, suggesting its potential used as a therapeutic agent to halt OA progression. Our strategy provided a new idea on the engineered stem cell therapy for OA.

The effect of the macrophage inflammatory response in the OA process has been proven very significant, and has been identified as a potential target to alleviate OA.^[^ ^20^ ^]^ Synovial inflammation (synovitis) is characterized by increased M1 polarization macrophages.^[^ ^21^ ^]^ However, the molecular mechanism underlying the sustained and indelible inflammation remains unclear. The voltage‐gated potassium channel Kv1.3 was first described in T cells.^[^ ^22^ ^]^ Recent studies have identified that Kv1.3 also exists in B lymphocytes, macrophages, retinal ganglion cells, microglia, and neurons.^[^ ^23^ ^]^ Furthermore, Kv1.3 expression in microglia is upregulated during the progression of Alzheimer's disease (AD) and Parkinson's disease and has been proposed as a potential therapeutic target for mitigating AD‐associated neuroinflammation.^[^ ^7^, ^24^ ^]^ The normal synovium consists of two major cell types: macrophages and fibroblast‐like synoviocytes.^[^ ^6a^ ^]^ In the progression of OA, multiple types of immune cell infiltration in the synovial, but the monocyte/macrophage cluster showed the most significant changes following injury, detected mouse samples including cartilage, synovia, synovium, infrapatellar fat pad, etc.^[^ ^25^ ^]^ Macrophage accumulation in the inner surface of synovial membrane pathologically, where the macrophage is the major type of immune cell in OA different from rheumatoid arthritis major in T cells.^[^ ^6b^ ^]^ Thus, our study demonstrated that blocking Kv1.3 channels in immune cells indeed has great potential to treat inflammatory diseases like OA. Therefore, we took measures to inhibit Kv1.3 in macrophages to treat OA. However, it does be worthy of study on the effect of multiple types of infiltrated immune cells later. Interestingly, the similar concentration of ShK(100 nm) on RAW264.7 cells is effective while the little effect on the C28/l2 chondrocyte cell line. It is an attractive question of what causes different effects in two types of cells responding to the same treatment. Maybe, different cells have different sensitivities and the macrophages are more sensitive, which is another meaningful problem to explore in the future.

During past decades, stem cell therapy including UCMSCs has been used to treat OA and got advanced effects in pre‐clinical models despite varying with each individual in early‐phase clinical trials.^[^ ^26^ ^]^ It has been proved that MSCs could produce many kinds of anti‐inflammatory cytokines, such as IL‐10, TGFβ, TSG‐6, and so on, which inhibit NF‐κB signaling pathways to reduce the generation of TNF‐α, IL‐1β, and other inflammatory mediators.^[^ ^27^ ^]^ However, the function of MSCs is limited especially in the local inflammatory environment in OA. Current novel delivery systems such as engineered microparticles are difficult to achieve clinical translation owe to their limited biocompatibility and biodegradability. Therefore, it is reasonable to explore new carriers with better biocompatibility to enhance their anti‐inflammatory ability in OA environment. The collection of UCMSCs from donors isnoninvasive, mature, and standardized. UCMSCs could proliferate in vitro for more than 10 generations without differentiated treatment, which is easy for amplification. Furthermore, it is easy to store UCMSCs because they have good freeze–thaw properties in liquid nitrogen within several cell generations, and thawed when transgenically engineering experiments are needed.^[^ ^28^ ^]^ UCMSCs show low immunogenicity and better biocompatibility for not inducing the proliferation of xenogeneic and allogeneic immune cells.^[^ ^29^ ^]^ Some studies have shown that injected stem cells could exist in the joint cavity for several weeks, indicating that MSCs could be a suitable delivery to carry and release drugs.^[^ ^30^ ^]^ Compared with previous studies on engineered MSCs, our research showed that ShK‐modified UCMSCs exhibited better therapeutic effects than single ShK administration, evidenced by longer dwell time and sustained release, which could reduce the frequency of invasive operations and risk of infection for patients. In our results, the fluorescence signal from UCMSCs pretreated with fluorochrome DiR could last for ≈6–7 days detected by a small animal imaging system, which is consistent with the result of the adipose‐derived stem cells from a previous study.^[^ ^30b^ ^]^ Additionally, in a clinical trial compared different doses, divided into low (2×10^6^), middle (10×10^6^), and high (50×10^6^) groups, patients treated with low‐dose ASCs (2×10^6^) improved best in pain and function compared with baseline.^[^ ^31^ ^]^ More adverse reactions like swell and pain occurred in the high dose group (150× 10^6^).^[^ ^32^ ^]^ However, another researcher found that higher dose group ADMSCs (100× 10^6^) achieved better pain relief and cartilage repair.^[^ ^33^ ^]^ The doses and time points of MSCs deserve further study in the preclinical trials in the future.

Along with the effective delivery vehicle, loaded medicine is also crucial. Many biologics and delivery vectors are immunogenic and the resulting neutralizing antibodies might weaken the effect of treatment. However, ShK is highly homologous to a domain of MMP23,^[^ ^34^ ^]^ and is likely recognized as components of their own cells by the immune system. As a result, ShK and its analogs elicit minimal or little immunogenicity in both murine and human models.^[^ ^35^ ^]^ According to our result viewed from H&E sections of the knee joint locally and major organs systemically, ShK from modified UCMSCs results little or negligible immunoreaction if any. While potential risks of using genetically modified stem cells in human patients still warrant attention, including production, proliferation, storage, and application personally.

By transfecting UCMSCs with lentivirus to overexpress ShK, we acquired UCMSCs with the ability to secrete functional ShK. The advantages of the ShK‐modified UCMSCs are low immunogenicity, longer dwell time, sustained drug release, and reduced invasive operations. In some chronic diseases, especially in OA, long‐term and repetitive injections could reduce patient compliance and increase the risk of infection. Our strategy provided a new idea on engineered stem cell therapy for chronic degenerative diseases. Nevertheless, limitations on our research remain to be optimized, such as the lack of experiments on large mammals. Secondly, it hangs in doubt what effect the Kv1.3 channel has in fibroblast‐like synoviocytes, chondrocytes, and cells from infrapatellar fat pad due to no targeting modification in ShK. Thirdly, the doses and time points of MSCs deserve further study in the preclinical trials in the future.

## 4 Conclusion

In conclusion, we developed engineered UCMSCs capable of secreting functional ShK that ameliorates OA by inhibiting M1 polarization efficiently. Inhibition of Kv1.3 decreases ROS production and M1 polarization via PI3K /Akt pathway in macrophages. Specifically, ShK‐UCMSCs have unique advantages, such as longer dwell time and sustained release which do not lead to adverse effects on main organs. Hence, our study provided a promising strategy for delivering peptide‐based drugs that can be used for treating more chronic inflammatory diseases.

## 5 Experimental Section

### Clinical Samples

The human synovial tissues were acquired from participants at the Affiliated Nanjing Drum Tower Hospital of Nanjing University Medical School. The OA group included 3 OA samples and 3 non‐OA samples. The samples were immediately immersed in 4% paraformaldehyde after surgery operation for later histological analysis. The research was approved by the Ethics Committee of Nanjing Drum Tower Hospital, and written informed consent was obtained from all patients (ethical approval number: 2020‐191‐02).

### Mice

Wild‐type C57BL/6 male mice were purchased from the Model Animal Research Center of Nanjing University. The surgical destabilization surgery of the medial meniscus (DMM) was performed according to a previous description.^[^ ^36^ ^]^ All animal experiments were approved by the Ethics Committee and the Institutional Animal Care and Use Committee of Drum Tower Hospital, Nanjing University Medical School (ethical approval number: 2021AE04013). The total intra‐articular injection was 10 µL PBS containing 1000 cells.^[^ ^37^ ^]^

### Cell Culture

The human umbilical cord mesenchymal stem cells (UCMSCs) were offered by the Clinical Stem Cell Center in the Affiliated Drum Tower Hospital, Medical School of Nanjing University, and confirmed before use to be positive for specific surface marker expressions.^[^ ^38^ ^]^ UCMSCs were cultured with DMEM/F12 (10565018, Gibco, USA) containing 10% v/v FBS (Gibco, USA) at 37 °C humidified air with 5% CO_2_. The cells were subcultured using trypsin (0.25%‐EDTA, 25‐043‐EL, WISENT, China) at 90% confluence for subsequent experiments.

The ATDC5 cell line (ZQ0938, Zhong Qiao Xin Zhou Biotechnology) and RAW264.7 cell line (CL‐0190, Procell Life Science & Technology) were cultured in DMEM (319‐005‐CL, WISENT) added with 10% FBS (FND500, ExCell Bio) and 100U mL^−1^ penicillin and 0.1 mg mL^−1^ streptomycin at 37 °C humidified air with 5% CO_2_. The concentration of LPS used to treat RAW264.7 cells was 100 ng mL^−1^ (LPS, MedChemExpress, Shanghai, China) for 24h. 100 nm ShK (QYAOBIO, Shanghai, China) was added to inhibit Kv1.3 to assess the role of Kv1.3 channels in M1 macrophage polarization.^[^ ^7d^ ^]^ The concentration of recombinant mouse IL‐1𝛽 protein (RP01340, ABclonal) used for ATDC5 cells was 10 ng mL^−1^ for 24 h to induce inflammation response.^[^ ^39^ ^]^

### Transfection

For lentiviral transduction, the ShK overexpressing lentivirus SmK‐ZsGreen‐PURO‐LUC purchased from Hanbio Biotech (Shanghai, China) was used to acquire stable human UCMSCs. The cells were transduced with the lentivirus (MOI = 100) in the presence of 6 µg mL^−1^ polybrene (MCE, Shanghai, China) for 72 h. Next, the transduced cells were selected by treatment with 2.5 µg ml^−1^ puromycin (MCE, Shanghai, China) for 24–48 h. Then exchange the medium with normal medium to culture until cell density of 90% for subsequent experiments.

### Statistical Analysis

All statistical analyses were performed using the GraphPad Prism software 8.4 and the data were presented as means ± SDs. The Student's *t*‐test was employed to analyze the differences between two groups and the ANOVA test was used for assessing the differences among multiple groups. The *p*‐value < 0.05 was considered significant in all experiments and showed in figures or legends as ^***^ *p* < 0.001; ^**^ *p* < 0.01; ^*^ *p* < 0.05, ns (no statistical difference).

## Conflict of Interest

The authors declare no conflicts of interest.

## Author Contributions

W.S.W. and X.Y.A. contributed equally to this work. W.S.W. and X.Y.A. conducted the majority of the assays, acquired, and analyzed data, and drafted the manuscript. L.L. assisted in project design and provided technique support. W.G., L.Y., N.L., and B.L. participated in animal experiments. L.L., Q.J., and B.S.G. conceived, and designed the project, supervised experiments, and provided research resources. All authors approved the final version of this manuscript.

## Supporting information



Supporting Information

## Data Availability

The data that support the findings of this study are available in the supplementary material of this article.
