# Supplementary material for: ShK‐modified UCMSCs Inhibit M1‐Like Macrophage Polarization and Alleviate Osteoarthritis Progression via PI3K/Akt Axis
Source: Adv Sci (Weinh). 2024 Dec 25;12(9):2406822. doi: 10.1002/advs.202406822 (PMC11884619; doi:10.1002/advs.202406822)
Supplement: Supplementary file 1 — Supporting Information [file ADVS-12-2406822-s001.docx]

Supporting Information

**ShK-modified UCMSCs Inhibit M1-like Macrophage Polarization and Alleviate Osteoarthritis Progression via** **PI3K/Akt Axis**

*Wenshu Wu, Xueying An, Wang Gong, Lin Yang, Na Liu, Bin Liu, Baosheng Guo^*^, Qing Jiang^*^, Lan Li^*^*

**This file includes:**

**Supplementary Figures and Figure legends**

**Supplementary Tables**

**Supplementary Materials and Methods**

**Supplementary Figures and Figure legends**


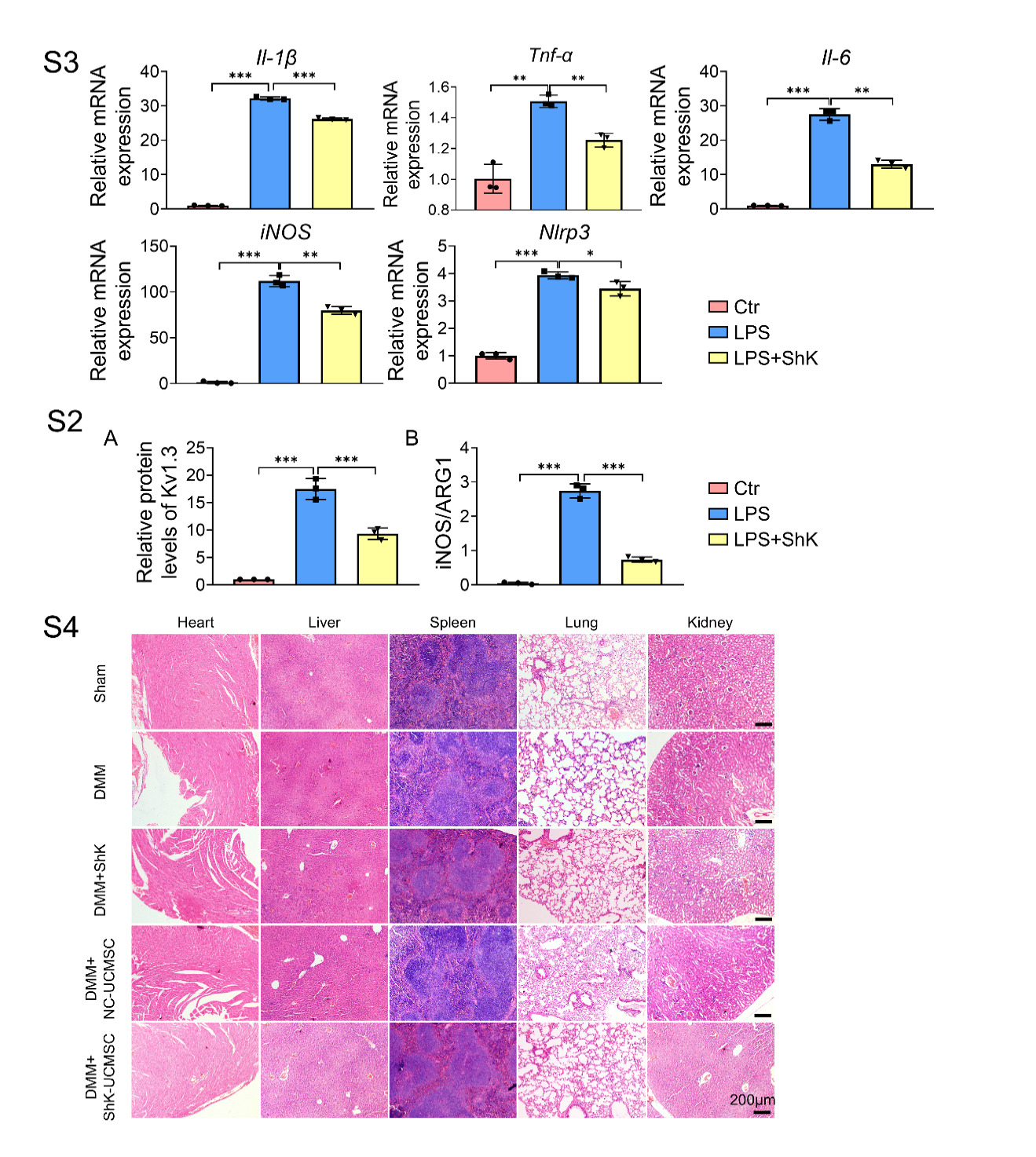


**Figure S1.** (A)Western blot and quantitative analysis of relative Kv1.3 protein level in RAW264.7 macrophages treated with LPS with or without ShK for 24 h. (B) Quantitative analysis of relative protein level ration of iNOS/ARG1 in RAW264.7 macrophages treated with LPS with or without ShK for 24 h. (n = 3) Data are means ± SD. Student’s two-tailed t test, ns, not significant p ≥ 0.05; *p < 0.05; **p < 0.01; ***p < 0.001.


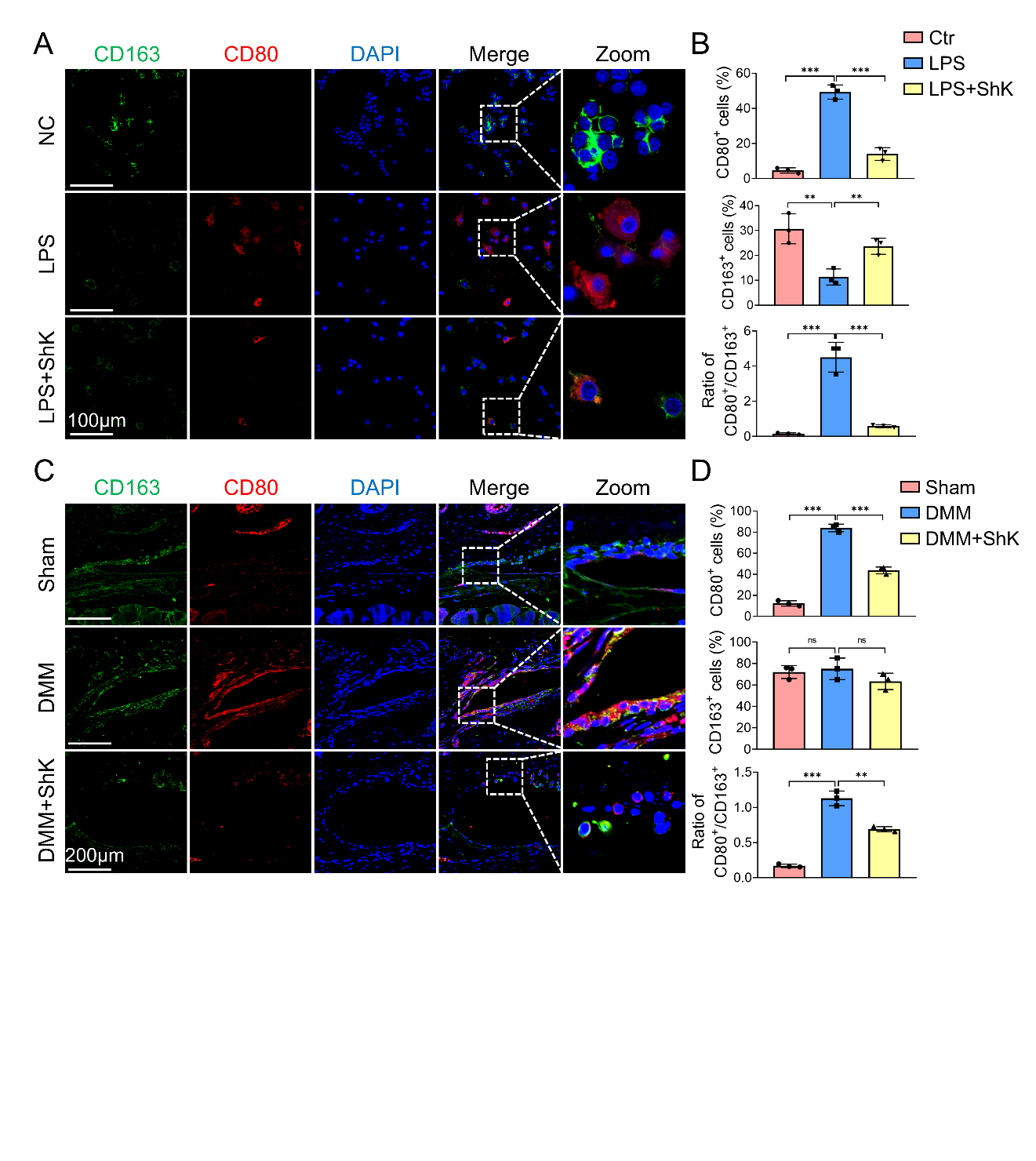


**Figure S2.** **Kv1.3 suppressor ShK effectively inhibits M1 macrophage polarization in vivo and in vitro.**

(A)Immunofluorescence images for M1 polarization marker CD80 (red) and M2 marker CD163 (green) in LPS induced RAW264.7 cells treated with or without ShK. Scale bar, 100 μm. (B) Quantitative analysis of CD80 and CD163 positive cells as a proportion of total cells RAW264.7 cells treated with or without ShK (n=3). (C) Representative immunofluorescence staining of CD80 and CD163 in 8-week sham or DMM groups. Scale bar, 200 μm. (D) Quantitative analysis of CD80 and CD163 positive cells in 8-week sham or DMM groups. (n = 3)

Data are means ± SD. Student’s two-tailed t test, ns, not significant p ≥ 0.05; *p < 0.05; **p < 0.01; ***p < 0.001.


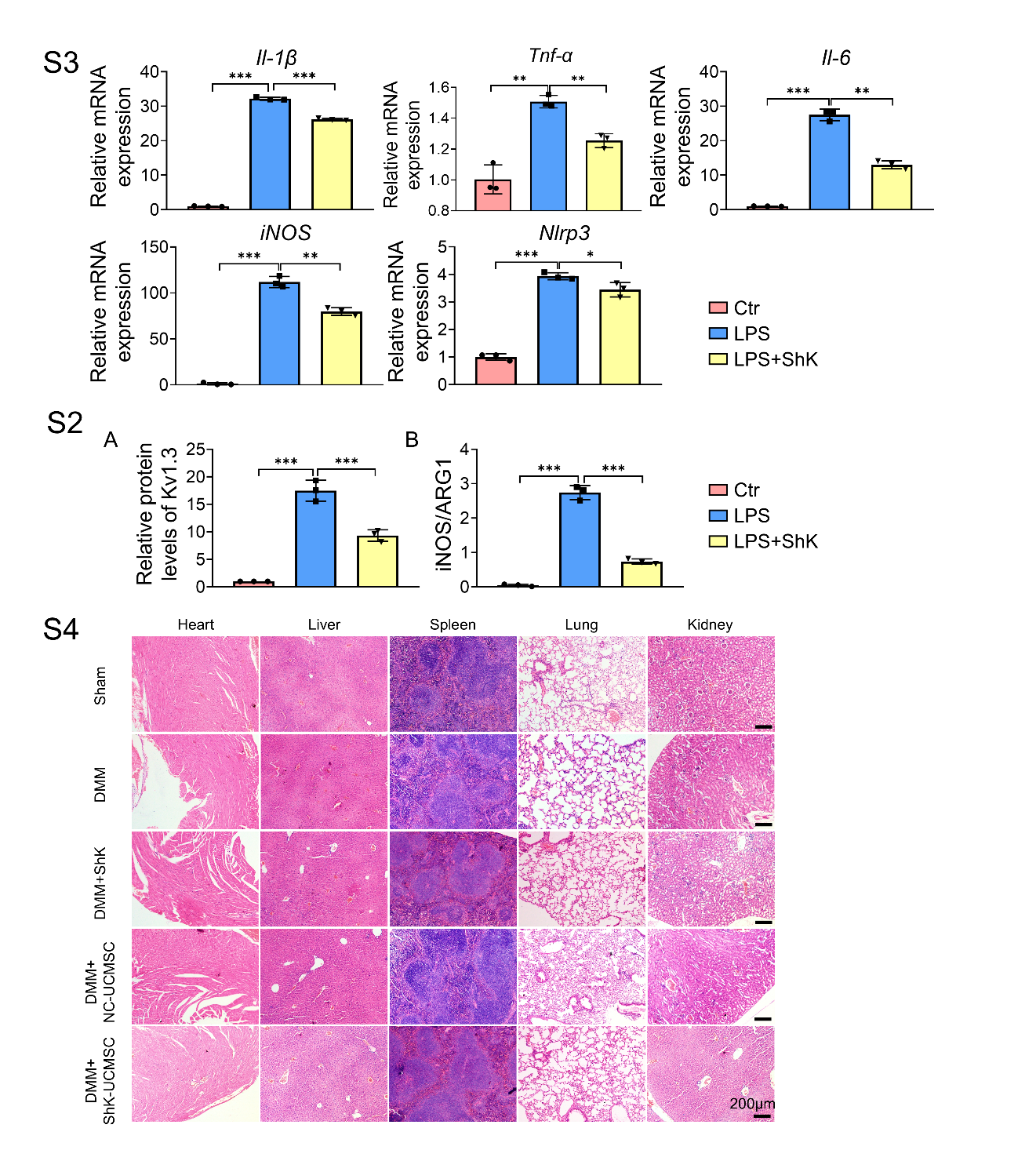


**Figure S3.** RT-qPCR for mRNA of inflammatory genes Il1b, Il6, Tnfα, Nlrp3 and Inos in macrophages treated with LPS with or without ShK for 24 h. (n = 3) Data are means ± SD. Student’s two-tailed t test, ns, not significant p ≥ 0.05; *p < 0.05; **p < 0.01; ***p < 0.001.

**
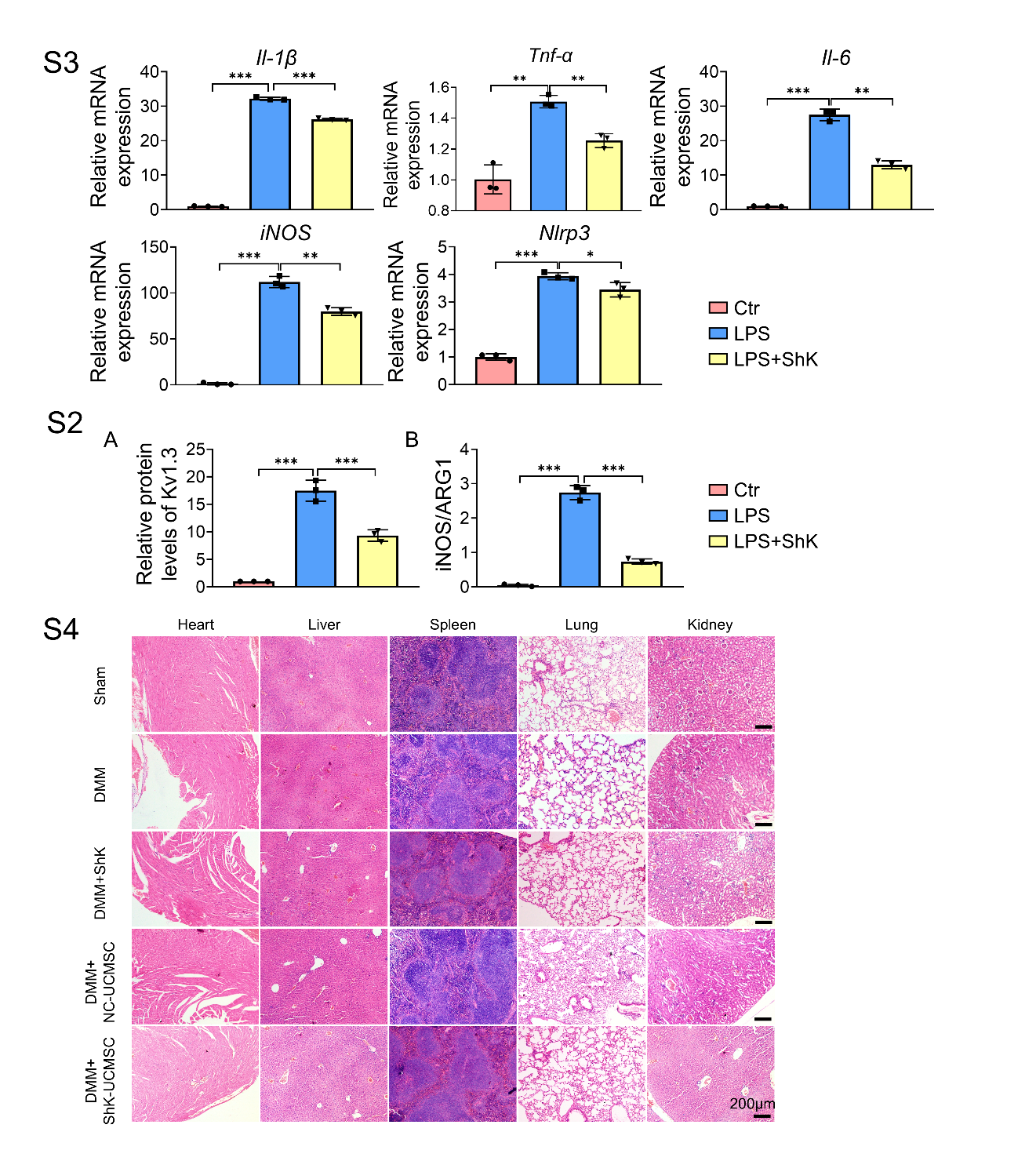
**

**Figure S4.** Representative H&E images of heart, liver, spleen, lung, kidney. Scale bar, 200 μm.


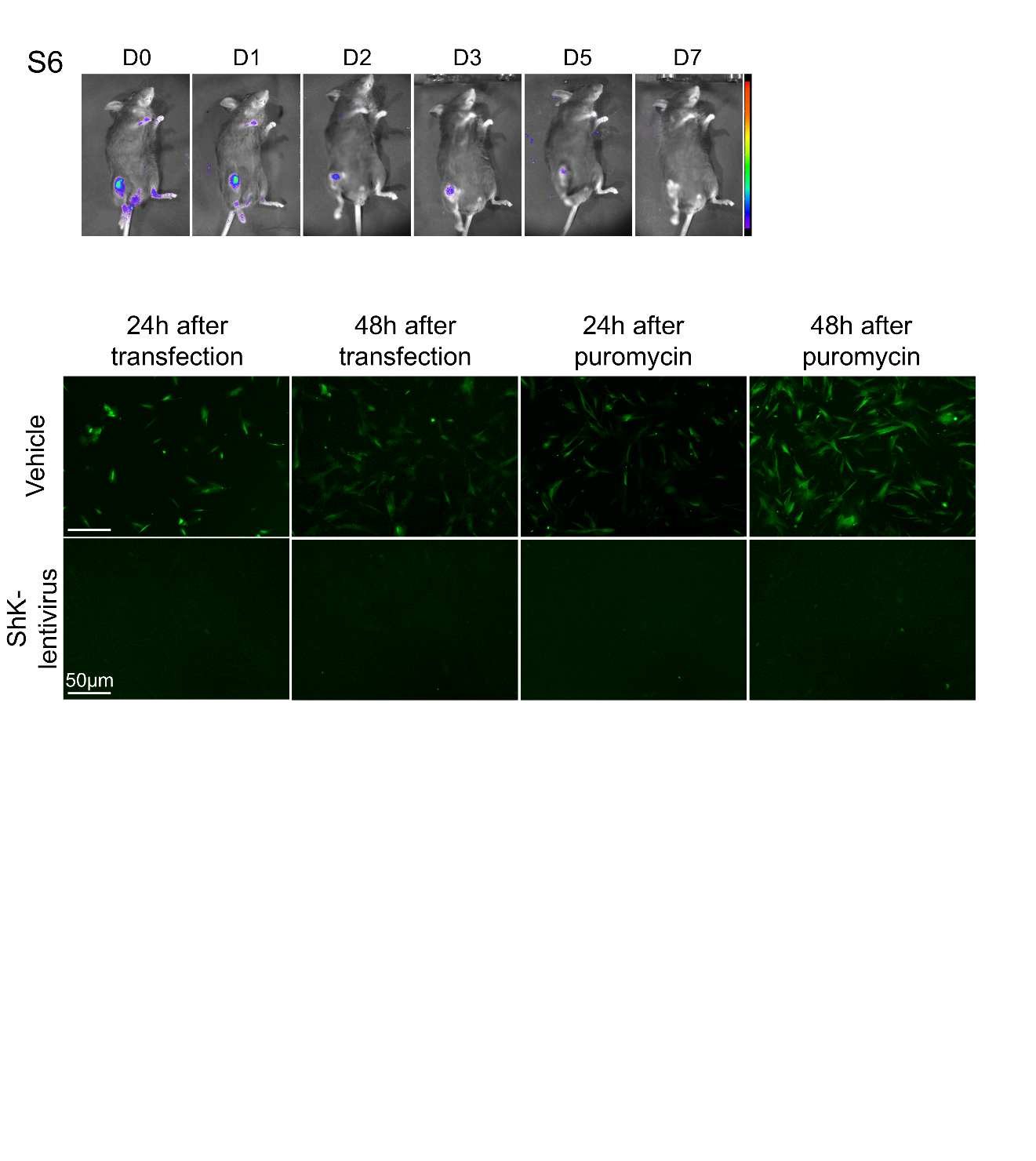


**Figure S5.** The procedure of UCMSCs transfected with lentivirus. The UCMSCs transfected with vehicle control lentivirus expressed green fluorescent protein (GFP), while the UCMSCs transfected with ShK overexpressing lentivirus did not. Scale bar, 100 μm.

**
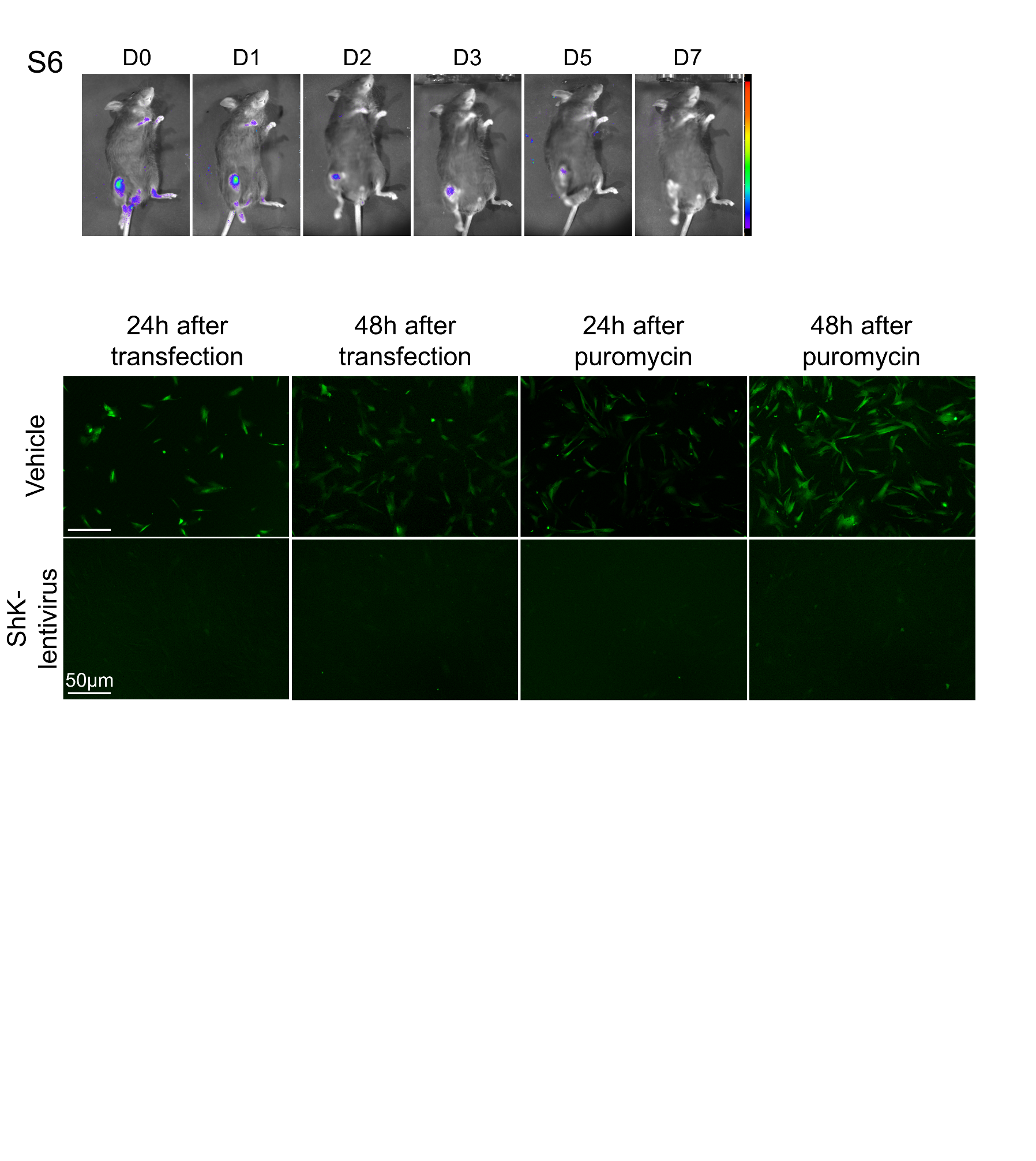
**

**Figure S6.** The fluorescence signal of DiR in keen joints from mice after intra-articular injection with Dir labeled UCMSCs.

**Supplementary Tables**

**Table S1**. Primers for quantitative real-time polymerase chain reaction (qRT-PCR) of mouse samples

| Target | Forward | Reverse |
| --- | --- | --- |
| *Il-1β* | TGCCACCTTTTGACAGTGATG | TGCCACCTTTTGACAGTGATG |
| *Tnf-α* | CCACCATCAAGGACTCAA | CAGGGAAGAATCTGGAAAGG |
| *iNos* | GCGCTCTAGTGAAGCAAAGC | AGTGAAATCCGATGTGGCCT |
| *Il-6* | TAGTCCTTCCTACCCCAATTTCC | TTGGTCCTTAGCCACTCCTTC |
| *Nlrp3* | ATTACCCGCCCGAGAAAGG | TCGCAGCAAAGATCCACACAG |
| *Actb* | GGCTGTATTCCCCTCCATCG | CCAGTTGGTAACAATGCCATGT |

**Supplementary Materials and Methods**

**Histology:** After fixed in 4% paraformaldehyde solution overnight, articular samples from harvested mice gone through decalcification with ethylenediaminetetraacetic acid (EDTA) for 15 days and the EDTA decalcification solution was changed daily. Then the samples were immersed in gradient ethanol and 100% xylene for dehydration and transparency. Next, paraffin embedded overnight, the samples were sectioned into 5 μm slices for H&E, Fast Green and Safranin O staining.

**Immunohistochemical (IHC) and immunofluorescence (IF) staining:** Briefly, the histological slides were deparaffinized in xylene and rehydrated into water after using ethanol ranging from 100 to 50%. The slides were incubated with pepsin buffer (G0142-100mL, Servicebio, Wuhan, China) for antigen retrieval, with 1% goat serum to block for 1-2 hour. Then, the slides were stained with primary antibodies at 4 °C overnight, including COL2A1 (BA0533) from BOSTER, MMP13 (18165-1-AP), CD80 (66406-1-LG), ARG1 (66129-1-lg) and CD163 (68218-1-lg) from Proteintech, iNOS (A3774) and COX2 (A1253) from ABclonal, Kv1.3 (GTX16679) from GeneTex, F4/80 (71299) from Cell Signaling Technology and Invitrogen (14-4801-81). Next day, the sections were washed with PBS and incubated secondary antibodies for 1 h. After washing 3 times by PBST, the sections were processed with DAB or antifade mounting medium including DAPI. IHC images were captured by the Olympus BX53 microscope, and IF images were acquired by a Leica DMi8 THUNDER or Olympus FV3000 system.

**Western Blot:** The protein of cells were collected by lysis buffer (Beyotime Biotechnology), added protease and phosphatase inhibitors (KeyGEN BioTech, Nanjing, China) on ice for 30 min. After denaturation at 99 ℃ for 10 min, the proteins gone through electrophoresis on SDS polyacrylamide gels, transferred to PVDF membranes (IPVH00010, Millipore) and blocked with blocking buffer (P0220, Beyotime Biotechnology) at room temperature for 1 h. The PVDF membranes were incubated with primary antibodies for COL2A1 (BA0533) from BOSTER, MMP13 (18165-1-AP), ARG1 (66129-1-lg) from Proteintech, Kv1.3 (GTX16679) from GeneTex, iNOS (A3774) and COX2 (A1253) from ABclonal overnight at 4°C. Next day, after washing 6 times, the membranes were incubated with HRP-conjugated secondary antibodies at room temperature for 1 h. Protein bands were visualized using ECL solution (Affinity, USA) by a chemiluminescence visualizer system (Tanon, Shanghai, China) and quantified by the Fiji software.

**Enzyme-linked Immunosorbent Assay (ELISA):** The ELISA kits included the IL-1𝛽 (SEKM-0002, Solarbio, China), TNF-𝛼 and IL-6 (RK00027 and RK00008, ABclonal, China) were utilized to quantify inflammatory factor concentrations of the RAW264.7 cell culture supernatant following the manufacturer’s instructions.

**Quantitative Real-Time Polymerase Chain Reaction (qRT-PCR):** The total RNA from cells was extracted by Total RNA Extraction Reagent (R401-01, Vazyme, China) according to the manufacturer’s instructions. Equal amounts of mRNA were reversely transcribed to cDNA by HiScript II qRT SuperMix for qPCR (+gDNA wiper) (Vazyme, Nanjing, China). ChamQTM SYBR Color qPCR Master Mix (Vazyme, Nanjing, China) was mixed with cDNA and gene-specific primers and performed the qPCR on a ViiA7 Fast Real-time PCR System (Applied Biosystems, Waltham, Massachusetts, USA). The relative expression levels were analyzed via the 2^−ΔΔCt^ method and normalized to the endogenous reference gene β-actin.

**Live & Dead Staining Assay:** The Live & Dead Viability/Cytotoxicity Assay Kit (KeyGEN BioTech, Nanjing, China) was utilized to distinguish live cells and dead cells by Calcein AM and propidium iodide (PI). Briefly, the AM and PI staining solution is diluted by dyeing solution at a ratio of 1:2000. After incubation for 20-30 min at 37 °C in the dark, the wells were washed three times with PBS. Then the images were captured by a fluorescence microscope (Nikon, Tokyo, Japan).

**ROS assay:** Supplement the medium with 10 mM fluorescent probe DCFH-DA (Beyotime, Shanghai, China) for 30 min at 37 °C. After fixing with 4% paraformaldehyde, LX-2 or L-02 were counterstained with Hoechst 3342. Images were captured using a confocal microscope (Olympus, Tokyo, Japan). The mean fluorescent intensity was calculated using Image-Pro Plus 6.0 software. There were three replicates per group.

**Micro-X-Ray Computed Tomography (μCT):** After fixation by PFA overnight, the mouse knee joints were scanned at 70 kV, 114 μA, and a resolution of 15.6 μm per pixel (VivaCT80; Scanco Medical AG, Switzerland). The 3D reconstruction images were acquired by Scanco Medical software.

**HPLC:** Gradient conditions were 35-85% B in 20 min at 1.0 mL/min (A buffer is 0.1% trifluoroacetic acid in water and B is acetonitrile containing 0.1% trifluoroacetic acid); detection is at 220 nm. The major product eluting at 8.5 min is the correctly peptide.
